# Liver Cirrhosis/Severe Fibrosis Is a Risk Factor for Anastomotic Leakage after Colorectal Surgery

**DOI:** 10.1155/2016/1563037

**Published:** 2016-12-26

**Authors:** Samuel Andreas Käser, Irina Hofmann, Niels Willi, Felix Stickel, Christoph Andreas Maurer

**Affiliations:** ^1^Department of General, Visceral, Vascular, and Thoracic Surgery, Hospital of Baselland, Rheinstrasse 26, 4410 Liestal, Switzerland; ^2^Department of Visceral and Transplant Surgery, University Hospital Zurich, Rämistrasse 100, 8091 Zurich, Switzerland; ^3^Institute of Pathology, Mühlemattstrasse 11, 4410 Liestal, Switzerland; ^4^Department of Gastroenterology, Hirslanden-Clinic Beau-Site, Schänzlihalde 1, 3000 Bern-25, 3013 Bern, Switzerland; ^5^Department of Surgery, Hirslanden-Clinic Beau-Site, Schänzlihalde 1, 3000 Bern-25, 3013 Bern, Switzerland

## Abstract

*Purpose*. Liver cirrhosis associated with high perioperative morbidity/mortality. This retrospective study determines whether liver cirrhosis represents a risk factor for anastomotic leakage after colonic anastomosis or not.* Methods*. Based on a prospective database with all consecutive colorectal resections performed at the authors' institution from 07/2002 to 07/2012 (*n* = 2104) all colonic and rectal anastomoses were identified (*n* = 1875). A temporary loop ileostomy was constructed in 257 cases (13.7%) either due to Mannheimer Peritonitis-Index > 29 or rectal anastomosis below 6 cm from the anal verge. More than one-third of the patients (*n* = 691) had postoperative contrast enema, either at the occasion of another study or prior to closure of ileostomy. The presence of liver cirrhosis and the development of anastomotic leakage were assessed by chart review.* Results*. The overall anastomotic leakage rate was 2.7% (50/1875). In patients with cirrhosis/severe fibrosis, the anastomotic leakage rate was 12.5% (3/24), while it was only 2.5% (47/1851) in those without (*p* = 0.024). The difference remained statistically significant after correction for confounding factors by multivariate analysis.* Conclusion*. Patients with liver cirrhosis/severe fibrosis have an increased risk of leakage after colonic anastomosis.

## 1. Introduction

How to deal with patients with known or unexpected liver cirrhosis remains a major challenge in colorectal surgery as liver cirrhosis bears a high risk of postoperative complications [1–5]. Although the morbidity and mortality in these patients have been studied [6–10], surprisingly little is known regarding the relation between liver cirrhosis and anastomotic leakage as the most feared complication after colorectal surgery.

Although the healing of an intestinal anastomosis has often been studied but remains poorly understood [11], there are several disturbances that might influence the anastomotic healing in patients with liver cirrhosis: first, portal hypertension with impaired regulation of splanchnic blood flow [12], second, the protein metabolism disorder [13], and, third, the immune dysfunction syndrome especially in presence of ascites [14]. Indeed factors correlated with adverse surgical outcome in patients with liver cirrhosis are higher intraoperative blood loss reflecting portal hypertension, hypalbuminemia reflecting protein metabolism disorder, and the presence of ascites [15]. Furthermore the severity of liver disease correlates with postoperative morbidity and mortality [16].

To our knowledge only animal models have shown a relationship between liver cirrhosis and anastomotic leakage so far [17]. This study determines, for the first time, whether liver cirrhosis is a risk factor for anastomotic leakage after colorectal surgery or not.

## 2. Methods

Based on an existing prospective colorectal database with all consecutive colorectal operations resections, all colonic and rectal anastomoses performed at the authors' institution from 07/2002 to 07/2012 were retrospectively identified (*n* = 1875). Regardless of the localization of the anastomosis an end-end anastomosis was always performed. In the case of rectal anastomosis double stapling technique using a circular stapler with a diameter of 33 mm was used and coloanal anastomosis was done with a single layered single-stitch suture with polydioxanone USP 5-0, while in the case of colonic anastomosis a hand-sewn anastomosis was done using a continuous double-layered suture with polydioxanone USP 5-0.

The policy on when to construct a temporary loop ileostomy (*n* = 257 cases, 13.7%) was as follows: Mannheimer Peritonitis-Index [18] higher than 29 and/or low rectal anastomosis up to 6 cm from anal verge.

The diagnosis of liver cirrhosis/severe fibrosis was based on the finding of a typical nodular surface of the liver during surgery and/or on liver biopsy. Histologically staging of fibrosis/cirrhosis was performed using the Ishak scoring system which defines cirrhosis for stages 5 and 6 and advanced fibrosis in stage 4 [19].

The development of anastomotic leakage and the risk factors were retrospectively assessed based on chart review.

A planned contrast enema was done in more than one-third of the patients (*n* = 691) either at the occasion of another study (prospective) or prior to closure of temporary loop ileostomy. In the remaining patients a CT scan with contrast enema was only done in the case of the presence of signs of anastomotic leakage, such as abdominal pain, fever, and elevation of inflammation markers (e.g., CRP elevation after postoperative day 3).

**Figure 1:**
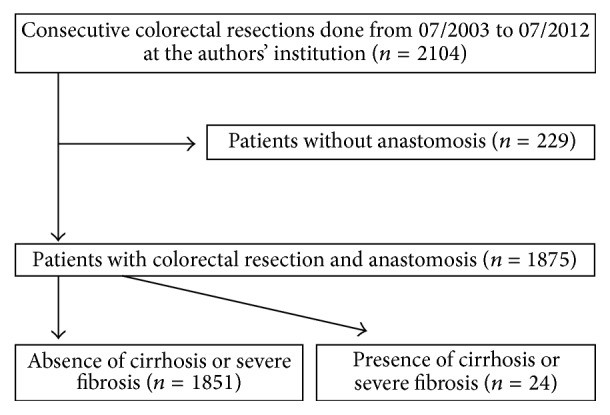
Flow diagram of the study methodology.

An anastomotic leak was defined as the extravasation of water-soluble contrast in the contrast enema or CT scan, a detected fluid collection (containing air bubbles and/or surrounded by a wall with contrast enhancement), fecal abdominal drainage, the intraoperative finding of anastomotic leakage, or the combination of two or more of these factors. An overview of the study methodology is presented in Figure 1.

### 2.1. Statistics

Results are expressed as median and range or mean and standard deviation whenever appropriate. Categorical data was analyzed with the two-sided Fisher's exact test. Continuous data was analyzed with the Wilcoxon rank sum score. Multivariate analyses were done by logistic regression. *p* values < 0.05 were considered statistically significant.

### 2.2. Ethics Statement

This retrospective study was approved by the ethical committee of northwestern and central Switzerland (EKNZ Number BASEC 2016-01311).

## 3. Results

The overall anastomotic leakage rate was 2.7% (50/1875). Among patients who developed an anastomotic leakage *n* = 6 (12%) were asymptomatic and only detected by imaging, *n* = 8 (16%) were managed conservatively, and *n* = 36 (72%) required revision laparotomy.

**Figure 2:**
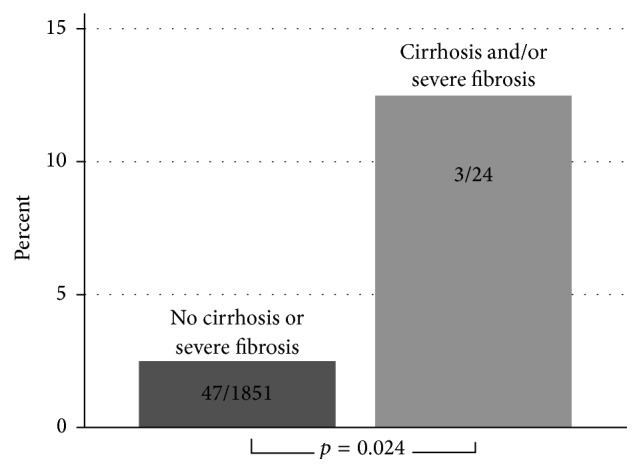
Anastomotic leak rate in absence versus in presence of liver cirrhosis and/or severe fibrosis.

The patients' characteristics are shown in Table 1 and the results of the univariate analyses of the risk factors for an anastomotic leak are shown in Table 2. The significantly higher leak rate in the case of presence of liver cirrhosis/severe fibrosis is shown in Figure 2.

Table 3 shows the results of the multivariate analyses where only male gender, lower albumin level, intake of immunosuppressive drugs, and the presence of severe fibrosis/cirrhosis remained statistically significant predictors of the development of an anastomotic leak after colonic surgery.

### 3.1. Subgroup Analyses

Patients with liver cirrhosis/severe fibrosis with anastomotic leakage revealed Child-Pugh-Turcotte scores A (*n* = 1) and B (*n* = 2), while those without anastomotic leakage had the following Child-Pugh-Turcotte scores A (*n* = 14), B (*n* = 4), and C (*n* = 1); *n* = 2 missing data. Patients with Child B or C score had no significantly higher anastomotic leakage rate after colorectal surgery than those with Child A score (*p* = 0.227).

From *n* = 79 patients with colorectal surgery without performed anastomosis *n* = 72 patients had sphincter saving procedures, while *n* = 7 had nonsphincter saving procedures. The indications for surgery were *n* = 26 perforated sigmoid diverticulitis, *n* = 14 anastomotic leakage, *n* = 7 colon ischemia, *n* = 8 colonic perforation of other origin, *n* = 11 colorectal cancer, *n* = 8 noncolorectal cancer, *n* = 3 chronic inflammatory bowel disease, *n* = 1 rectal prolapse, and *n* = 1 perianal abscess. The rate of liver cirrhosis in these patients was not significantly higher (2.5%, 2/79) than in the population with anastomosis performed (1.3%, 24/1875, *p* = 0.288).

From 71 patients with reversal of a colostomy only two patients had liver cirrhosis/or high-grade fibrosis. These two patients did not develop anastomotic leakage.

## 4. Discussion

Liver cirrhosis is a known major risk factor for postoperative complications in general and colorectal surgery with a reported morbidity of up to 50% and mortality up to 25%. The severity of the disease correlates with postoperative morbidity and mortality [16]. Surprisingly little is known about the connection between liver cirrhosis and anastomotic leakage after colorectal surgery [17]. Nevertheless surgeons dislike or even avoid performing colonic or rectal anastomosis in patients with liver cirrhosis.

The present study aimed to determine if liver cirrhosis/severe fibrosis represents a risk factor for leakage after colorectal surgery or not. Indeed the results support the assumption that cirrhosis/severe fibrosis is a significant risk factor for the development of an anastomotic leak after colorectal surgery.

The presented data complies with earlier reports showing that cirrhotic patients are in an immunocompromised state with a high risk for bacterial translocation and septic conditions [14, 20]. As in other studies hypalbuminemia as a marker of liver dysfunction was shown to be associated with anastomotic leakage [21–23].

Which measures can be taken to lower the risk of anastomotic leakage in colorectal surgery in patients with liver cirrhosis?

First, pharmacotherapy of patients with liver cirrhosis should be optimized prior to surgery, particularly regarding portal hypertension [24]. For the latter, appropriate treatment with nonselective beta-blockers should be implemented. For those nonresponsive to medical therapy, portal hypertension can be lowered by inserting a transjugular intrahepatic portosystemic shunt [25]. However, this is limited to elective procedures and the impact on postoperative outcome is yet unclear [26].

During operation the sequels of anastomotic leakage can be lowered by construction of a temporary loop ileostomy. But the risk for complications after construction of temporary loop ileostomy (peristomal leaking, infection, peristomal eventration, bleeding from peristomal varices, and complications related to stoma closure) seems to be elevated in patients with liver cirrhosis as well [6, 27]. Thus the role of a temporary loop ileostomy in colorectal surgery in the presence of liver cirrhosis and/or severe fibrosis remains open.

The diagnosis of liver cirrhosis/severe fibrosis was defined as typical nodular surface of the liver described by the surgeon and/or histological diagnosis of liver cirrhosis/severe fibrosis by liver biopsy. Due to possible sampling error of liver biopsy in liver cirrhosis about one-third of the diagnoses are missed by biopsy only [28]. The intraoperative typical nodular surface is not an unambiguous marker of liver cirrhosis as it can be absent in about 1% of the patients with histologically confirmed liver cirrhosis [28] and it can be present in patients with severe fibrosis [29]. Doing both laparoscopy/laparotomy and liver biopsy improves the diagnostic yield up to 98% [30]. Thus, we feel that the definition of liver cirrhosis/severe fibrosis in the present study is justified. Furthermore it fits the real world, as during surgery surgeons sometimes have to deal with an unexpected nodular surface of the liver. However, for future, especially prospective studies, noninvasive methods such as transient elastography or shear-wave elastography potentially allow for improved risk stratification.

### 4.1. Limitations

The main drawback of this study is its retrospective design. However, we tried to minimize a potential bias by including all consecutive patients registered in a prospective database who had colorectal resection with or without anastomosis at the same institution in a predefined period of ten years. No selection bias due to not performing colonic or rectal anastomosis in patients with liver cirrhosis could be detected.

Although the study has some limitations, we feel that the results of this study are valid and that colorectal surgeons should be aware of the higher risk of anastomotic leakage in patients with liver cirrhosis or high-grade fibrosis.

Clearly, to confirm the results of the present study a prospective study should be performed.

## 5. Conclusion

Patients with liver cirrhosis or severe fibrosis have an increased leak rate after colonic anastomosis.

## Figures and Tables

**Table 1:** Patients' characteristics.

Risk factor	Liver cirrhosis/severe fibrosis (total *n* = 24)	No liver cirrhosis/severe fibrosis (total *n* = 1851)	*p* value
Mean age (SD)	65.5 (11.6)	65.0 (14.4)	0.943
Male gender	17 (71%)	892 (48%)	0.039
Median ASA score (range)	3 (2–4)	2 (1–5)	0.002
Cardiac comorbidities	19 (79%)	818 (44%)	0.008
Vascular comorbidities	3 (13%)	176 (10%)	0.737
Diabetes	6 (25%)	157 (8%)	0.022
Pulmonary comorbidities	9 (38%)	224 (12%)	0.003
Tobacco abuse	7 (29%)	269 (15%)	0.094
Obesity	18 (75%)	948 (51%)	0.086
Dementia	3 (13%)	20 (1%)	0.004
Immunosuppressive drugs	1 (4%)	83 (4%)	1.000
Nonsteroidal anti-inflammatory drugs	4 (17%)	248 (13%)	0.770
Presence of cancer	11 (46%)	537 (29%)	0.131
Presence of peritonitis	4 (17%)	173 (9%)	0.294
Large bowel obstruction	1 (4%)	103 (6%)	1.000

SD: standard deviation; ASA: American Society of Anesthesiologists.

**Table 2:** Results of the univariate analyses of the risk factors of anastomotic leakage after colonic anastomosis.

Risk factor	Anastomotic leak (*n* = 50)	No anastomotic leak (*n* = 1805)	*p* value
Mean age (SD)	68.8 (14.9)	64.9 (14.4)	0.028
Male gender	*n* = 33	*n* = 876	0.021
Median ASA score (Range)	2 (1–4)	2 (1–5)	0.156
Cardiac comorbidities	*n* = 28	*n* = 810	0.322
Vascular comorbidities	*n* = 5	*n* = 174	1.000
Diabetes	*n* = 10	*n* = 153	0.022
Pulmonary comorbidities	*n* = 9	*n* = 224	0.396
Tobacco abuse	*n* = 7	*n* = 269	0.437
Obesity	*n* = 28	*n* = 938	1.000
Dementia	*n* = 2	*n* = 21	0.142
Immunosuppressive drugs	*n* = 7	*n* = 77	0.010
Nonsteroidal anti-inflammatory drugs	*n* = 7	*n* = 245	1.000
Presence of cancer	*n* = 16	*n* = 532	1.000
Presence of peritonitis	*n* = 5	*n* = 172	1.000
Large bowel obstruction	*n* = 3	*n* = 101	1.000
Liver cirrhosis or severe fibrosis	*n* = 3	*n* = 21	0.024
Mean (SD) white blood cell count (1/10*E*9)	8.5 (3.8)	8.2 (4.0)	0.300
Mean (SD) haemoglobin level	12.7 (2.4)	13.3 (2.0)	0.084
Mean (SD) sodium level	138.9 (4.2)	140.0 (3.1)	0.471
Mean (SD) potassium level	4.1 (0.5)	4.1 (1.1)	0.757
Mean (SD) creatinine level	88.4 (48)	79.6 (43.6)	0.131
Mean (SD) albumin level	37.6 (4.8)	40.2 (5.7)	<0.001

SD: standard deviation; ASA: American Society of Anesthesiologists.

**Table 3:** Results of the multivariate analyses of the detected risk factors for anastomotic leakage after colonic anastomosis.

Risk factor	Odds ratio	95% confidence interval	*p* value
Male gender	2.3	1.1–4.4	0.021
Age	1.0	1.0–1.1	0.422
Diabetes	1.4	0.6–3.5	0.439
Albumin level	1.0	0.9–1.0	0.042
Immunosuppressive drugs	2.9	1.0–7.9	0.045
Liver cirrhosis or severe fibrosis	4.1	1.1–15.5	0.036

ASA: American Society of Anesthesiologists.
